# Editorial: New insights into atypical Alzheimer's disease: from clinical phenotype to biomarkers

**DOI:** 10.3389/fnins.2024.1414443

**Published:** 2024-04-30

**Authors:** Neha Atulkumar Singh, Irene Sintini

**Affiliations:** ^1^Department of Neurology, Mayo Clinic, Rochester, MN, United States; ^2^Department of Radiology, Mayo Clinic, Rochester, MN, United States

**Keywords:** atypical Alzheimer's disease, posterior cortical atrophy, logopenic progressive aphasia, dysexecutive Alzheimer's disease, corticobasal syndrome

Alzheimer's disease (AD) is a neurodegenerative disease commonly associated with memory loss and is thought as a disease affecting the elderly (Frisoni et al., 2022). However, atypical clinical presentations can also occur, and they have been gaining interest in the past few decades, with research now focusing on investigating the underlying mechanisms and improving diagnosis of these atypical presentations of AD (Gorno-Tempini et al., 2008; Crutch et al., 2012; Townley et al., 2020; Ossenkoppele et al., 2022; Shir et al., 2023). Atypical AD presentations are said to account for at least 25% of all AD cases (Murray et al., 2011) and are characterized by the presence of initial and predominant deficits in non-memory domains, such as visual (Crutch et al., 2012), language (Gorno-Tempini et al., 2008), behavioral (Ossenkoppele et al., 2015b), executive (Townley et al., 2020), or motor (Shir et al., 2023) difficulties, and younger age at onset (Graff-Radford et al., 2021). Despite significant clinical and neuroimaging advancements, about half of these patients are misdiagnosed or receive a delayed diagnosis (Balasa et al., 2011) and they do not meet eligibility for AD clinical trials as being amnestic is a requirement in these trials. Hence, there is a need to raise awareness of these atypical AD phenotypes.

Through this Research Topic we intend to advocate that atypical AD phenotypes are valuable to the AD field and should be the focus of future research. We also propose treating these heterogenous patients as a part of the AD continuum, instead of considering them atypical. We present six articles that touch upon the concepts of the “heterogeneity in AD,” “atypical AD is a clinical spectrum” and “are atypical AD variants really atypical.”

Clinical presentations of AD are heterogenous and are associated with different neurodegenerative and neuropathological patterns, despite similar topographic distribution of amyloid. However, recent research has shown significant overlap across domains of clinical presentations, gray-matter atrophy, and tau deposition patterns in atypical AD phenotypes (Migliaccio et al., 2009; Owens et al., 2018; Singh et al., 2024). A study by Phillips et al. in this Research Topic also highlighted the heterogeneity and overlap in neuroimaging abnormalities across the AD continuum by investigating white-matter integrity and microstructure differences. In this study, they found both distinct and overlapping white-matter degeneration profiles across the AD continuum, involving key regions and fiber tracts, which mirrored the findings of previous studies on atypical AD employing various imaging modalities (Caso et al., 2015; Madhavan et al., 2016; Sintini et al., 2019; Singh et al., 2023). Phillips et al. effectively extended their study by assessing differences relative to typical AD patients and reporting a good correspondence between phenotypic white-matter differences and previously reported gray-matter atrophy. Therefore, gray-matter progression may partially be related to the white-matter degeneration seen in atypical AD phenotypes. Likewise, Mohanty et al. explored heterogeneity in atrophy across the full cognitive spectrum of amyloid positive individuals. They found atrophy-based severity to be strongly associated with tau burden and performance in executive and language domains, emphasizing the importance of language as a relevant cognitive sphere for testing across the AD continuum.

In complementary studies, Putcha et al., Rezaii et al., and Ahmed et al. highlighted the overlap in clinical features across atypical AD phenotypes. Putcha et al. investigated the immediate auditory naming performance in atypical AD patients, particularly the visual variant of AD. They assessed naming in the absence of a visual perceptual demand, which is inherent in most tests used to study anomia. More than half of the visual variant patients in their study demonstrated anomia and had a naming difficulty, which makes a valuable contribution to the emerging literature describing language deficits in the visual variant of AD (Putcha et al., 2018; Tetzloff et al., 2021; Singh et al., 2024). Rezaii et al. on a similar note explored the distinction in language performance between visually-dependent and visually-independent contexts. They proposed language as a sensitive diagnostic domain and suggested that the language abnormalities recently being identified in the visual variant of AD may be byproducts of visuospatial processing deficits. The current literature reports the spread of neurodegeneration beyond visual areas into regions involving language processing as a potential reason for language abnormalities in the visual variant (Migliaccio et al., 2009; Ossenkoppele et al., 2015a; Singh et al., 2024). However, Rezaii et al. offers an alternate theory by proposing that visuospatial deficits may hinder transfer of sensory information required for a modal language processing. Ahmed et al. furthered our understanding of language deficits in the visual variant of AD by investigating the language measures temporally. They reported longer preparation, utterance duration, silent pause duration, speech duration and notably longer time to initiate response in the visual AD variant. They effectively implied that there may be impaired interplay between linguistic and cognitive abilities, by presenting evidence of vulnerability in cognitive systems overseeing planning, execution, and attentional control mechanisms. Together these studies highlight how quickly the disease progresses into secondary domains and the importance of comprehensive clinical and neuropsychological assessments in atypical AD phenotypes.

To truly appreciate the significance of this Research Topic, one must understand how challenging and necessary it is to conduct research in atypical AD phenotypes. Whitwell's perspective article beautifully emphasizes these challenges, such as how most atypical AD patients are only diagnosed at specialized centers that employ a biomarker-based approach for evidence of AD. Despite detailed clinical characterization of these phenotypes, there is a lack of consideration when compared to the typical-amnestic presentation of AD. Although atypical AD patients meet eligibility for treatment with current AD therapeutics based on abnormal amyloid biomarkers, they are not commonly considered for AD clinical trials. Moreover, the phenotypic overlap across the variants of atypical AD creates further diagnostic challenges for categorical phenotyping. For these reasons, labeling these patients as “atypical” may have diminished their importance and value in the field and it may be better to think of them as AD with cognitive impairment that affects multiple domains.

In conclusion, this Research Topic contains a treasure-trove of information. These studies have collectively advanced our understanding and hold immense potential for informing future work in atypical AD phenotypes.

## Author contributions

NS: Conceptualization, Writing – original draft, Writing – review & editing. IS: Writing – original draft, Writing – review & editing.

## References

[B1] Balasa M. Gelpi E. Antonell A. Rey M. J. Sanchez-Valle R. Molinuevo J. L.. (2011). Clinical features and APOE genotype of pathologically proven early-onset Alzheimer disease. Neurology 76, 1720–1725. 10.1212/WNL.0b013e31821a44dd 21576687

[B2] Caso F. Agosta F. Mattavelli D. Migliaccio R. Canu E. Magnani G.. (2015). White matter degeneration in atypical Alzheimer disease. Radiology 277, 162–172. 10.1148/radiol.2015142766 26018810

[B3] Crutch S. J. Lehmann M. Schott J. M. Rabinovici G. D. Rossor M. N. N. C. (2012). Posterior cortical atrophy. Lancet Neurol. 11, 170–178. 10.1016/S1474-4422(11)70289-7 22265212 PMC3740271

[B4] Frisoni G. B. Altomare D. Thal D. R. Ribaldi F. Van Der Kant R. Ossenkoppele R.. (2022). The probabilistic model of Alzheimer disease: the amyloid hypothesis revised. Nat. Rev. Neurosci. 23, 53–66. 10.1038/s41583-021-00533-w 34815562 PMC8840505

[B5] Gorno-Tempini M. L. Brambati S. M. Ginex V. Ogar J. Dronkers N. F. Marcone A.. (2008). The logopenic/phonological variant of primary progressive aphasia. Neurology 71, 1227–1234. 10.1212/01.wnl.0000320506.79811.da 18633132 PMC2676989

[B6] Graff-Radford J. Yong K. X. X. Apostolova L. G. Bouwman F. H. Carrillo M. Dickerson B. C.. (2021). New insights into atypical Alzheimer's disease in the era of biomarkers. Lancet Neurol. 20, 222–234. 10.1016/S1474-4422(20)30440-3 33609479 PMC8056394

[B7] Madhavan A. Schwarz C. G. Duffy J. R. Strand E. A. Machulda M. M. Drubach D. A.. (2016). Characterizing white matter tract degeneration in syndromic variants of Alzheimer's disease: a diffusion tensor imaging study. J. Alzheimers Dis. 49, 633–643. 10.3233/JAD-150502 26484918 PMC10038690

[B8] Migliaccio R. Agosta F. Rascovsky K. Karydas A. Bonasera S. Rabinovici G. D.. (2009). Clinical syndromes associated with posterior atrophy: early age at onset AD spectrum. Neurology 73, 1571–1578. 10.1212/WNL.0b013e3181c0d427 19901249 PMC2777069

[B9] Murray M. E. Graff-Radford N. R. Ross O. A. Petersen R. C. Duara R. D. W. (2011). Neuropathologically defined subtypes of Alzheimer's disease with distinct clinical characteristics: a retrospective study. Lancet Neurol. 10, 785–796. 10.1016/S1474-4422(11)70156-9 21802369 PMC3175379

[B10] Ossenkoppele R. Cohn-Sheehy B. I. La Joie R. Vogel J. W. Moller C. Lehmann M.. (2015a). Atrophy patterns in early clinical stages across distinct phenotypes of Alzheimer's disease. Hum. Brain Mapp. 36, 4421–4437. 10.1002/hbm.22927 26260856 PMC4692964

[B11] Ossenkoppele R. Pijnenburg Y. A. Perry D. C. Cohn-Sheehy B. I. Scheltens N. M. Vogel J. W.. (2015b). The behavioural/dysexecutive variant of Alzheimer's disease: clinical, neuroimaging and pathological features. Brain 138, 2732–2749. 10.1093/brain/awv191 26141491 PMC4623840

[B12] Ossenkoppele R. Singleton E. H. Groot C. Dijkstra A. A. Eikelboom W. S. Seeley W. W.. (2022). Research criteria for the behavioral variant of Alzheimer disease: a systematic review and meta-analysis. JAMA Neurol. 79, 48–60. 10.1001/jamaneurol.2021.4417 34870696 PMC8649917

[B13] Owens T. E. Machulda M. M. Duffy J. R. Strand E. A. Clark H. M. Boland S.. (2018). Patterns of neuropsychological dysfunction and cortical volume changes in logopenic aphasia. J. Alzheimers Dis. 66, 1015–1025. 10.3233/JAD-171175 30372673 PMC6322407

[B14] Putcha D. Mcginnis S. M. Brickhouse M. Wong B. Sherman J. C. B. C. (2018). Executive dysfunction contributes to verbal encoding and retrieval deficits in posterior cortical atrophy. Cortex 106, 36–46. 10.1016/j.cortex.2018.04.010 29864594 PMC6120771

[B15] Shir D. Pham N. T. T. Botha H. Koga S. Kouri N. Ali F.. (2023). Clinicoradiologic and neuropathologic evaluation of corticobasal syndrome. Neurology 101, e289–e299. 10.1212/WNL.0000000000207397 37268436 PMC10382268

[B16] Singh N. A. Graff-Radford J. Machulda M. M. Carlos A. F. Schwarz C. G. Senjem M. L.. (2024). Atypical Alzheimer's disease: new insights into an overlapping spectrum between the language and visual variants. J. Neurol. 12, 1–16. 10.1007/s00415-024-12297-1 38551740 PMC11273322

[B17] Singh N. A. Graff-Radford J. Machulda M. M. Pham N. T. T. Schwarz C. G. Reid R. I.. (2023). Diffusivity changes in posterior cortical atrophy and logopenic progressive aphasia: a longitudinal diffusion tensor imaging study. J. Alzheimers Dis. 94, 709–725. 10.3233/JAD-221217 37302032 PMC10785680

[B18] Sintini I. Schwarz C. G. Martin P. R. Graff-Radford J. Machulda M. M. Senjem M. L.. (2019). Regional multimodal relationships between tau, hypometabolism, atrophy, and fractional anisotropy in atypical Alzheimer's disease. Hum. Brain Mapp. 40, 1618–1631. 10.1002/hbm.24473 30549156 PMC6615561

[B19] Tetzloff K. A. Duffy J. R. Strand E. A. Machulda M. M. Schwarz C. G. Senjem M. L.. (2021). Phonological errors in posterior cortical atrophy. Dement. Geriatr. Cogn. Disord. 50, 195–203. 10.1159/000516481 34274933 PMC8376759

[B20] Townley R. A. Graff-Radford J. Mantyh W. G. Botha H. Polsinelli A. J. Przybelski S. A.. (2020). Progressive dysexecutive syndrome due to Alzheimer's disease: a description of 55 cases and comparison to other phenotypes. Brain Commun. 2:fcaa068. 10.1093/braincomms/fcaa068 32671341 PMC7325839

